# A multi-component intervention to support breastfeeding in Lebanon: A randomized clinical trial

**DOI:** 10.1371/journal.pone.0218467

**Published:** 2019-06-14

**Authors:** Mona Nabulsi, Hani Tamim, Lama Shamsedine, Lama Charafeddine, Nadine Yehya, Tamar Kabakian-Khasholian, Saadieh Masri, Fatima Nasser, Soumaya Ayash, Diane Ghanem

**Affiliations:** 1 Department of Pediatrics and Adolescent Medicine, Faculty of Medicine, American University of Beirut, Beirut, Lebanon; 2 Biostatistics Unit, Clinical Research Institute, Faculty of Medicine, American University of Beirut, Beirut, Lebanon; 3 Olayan School of Business, American University of Beirut, Beirut, Lebanon; 4 Department of Health Promotion and Community Health, Faculty of Health Sciences, American University of Beirut, Beirut, Lebanon; 5 Faculty of Medicine, American University of Beirut, Beirut, Lebanon; University of Ghana, GHANA

## Abstract

**Background:**

Effective evidence-based breastfeeding support interventions can bolster breastfeeding practices. This study investigated the effect of a multi-component breastfeeding support intervention delivered in hospital and home settings on six-month exclusive breastfeeding (EBF) relative to standard care.

**Methods:**

This is a parallel group, randomized clinical trial, in which 362 healthy pregnant women with singleton pregnancy were randomly allocated to a multi-component intervention that included antenatal breastfeeding education, professional, and peer support, delivered in hospital and home settings for six months (experimental, n = 174), or to standard care (control, n = 188). The primary outcome was six-month EBF rate. Secondary outcomes were exclusive and any breastfeeding rates at one and three months, maternal breastfeeding knowledge, attitude, and behavior at six months, and satisfaction with the intervention.

**Results:**

The crude six-month EBF rate was similar in both groups (35.2% vs. 28.1% in the experimental and control groups, respectively, *p* = 0·16). In adjusted analysis, six-month exclusivity was twice as likely in the experimental group relative to standard care (OR = 2.02; *95%CI*: 1.20 to 3.39); whereas the odds for any breastfeeding were similar. Participants compliant with all three components were six times more likely to practice EBF for six months relative to standard care (OR = 6.63; *95% CI*: 3.03 to 14.51). Breastfeeding knowledge of the experimental group, at six months, was significantly improved compared to the control. No changes were observed in breastfeeding attitude or behavior.

**Conclusions:**

Combining education with peer and professional breastfeeding support improved six-month breastfeeding exclusivity and knowledge.

## Introduction

Breastfeeding is an important public health measure that impacts short-, and long-term outcomes of children and their mothers [1]. Evidence from systematic reviews reveals that breastfeeding is associated with reduced child under-five mortality, infections, and dental malocclusion; and with higher child intelligence quotient. Moreover, it reduces maternal risks of breast and ovarian cancers, and type 2 diabetes. It is estimated that scaling up of breastfeeding prevents 823,000 child deaths, and 20,000 maternal deaths from breast cancer each year [1]. Despite this overwhelming evidence in support of breastfeeding practice, breastfeeding rates of developing, as well as developed countries are disappointingly low. In low- and middle-income countries, only 37% of infants are exclusively breastfed until six months of age, with much lower rates reported from high-income countries. The prevalence of any breastfeeding at twelve months in low-income countries is less than 90%, and is less than 20% in high-income countries, with middle-income countries having in between rates [1]. The effectiveness of antenatal breastfeeding education, peer support, or professional lactation support has been demonstrated in several systematic reviews [2–8]. For example, peer support reduced the risk of not breastfeeding by 30% in low- or middle-income countries, and by 7% in high-income countries [2], and all forms of extra support, analysed together, increased the duration of exclusive breastfeeding (EBF) until six months [3,4]. Breastfeeding education increased initiation rates in low-income USA women, as compared to standard care [4], and breastfeeding promotion interventions improved six-month EBF rates of developing countries by six fold [5].

Lebanon, an upper middle-income country, has low exclusive breastfeeding and continuation rates. While initiation rate is high at 96% [9], six-month exclusive breastfeeding drops to 2%, one of the lowest in the region [10]. Barriers to breastfeeding include maternal and community misconceptions about breastfeeding, lack of professional lactation support, failure to implement national policies that promote and protect breastfeeding practices, lack of social support especially at the family level, as well as other socio-demographic factors [11,12]. Moreover, hospitals and maternities in Lebanon do not comply with the World Health Organization’s (WHO) ten steps of Baby Friendly Hospital Initiative [13], and health professionals who care for nursing mothers lack the WHO and UNICEF recommended training in the prevention and treatment of breastfeeding problems [14,15]. This randomized trial was conducted to address these challenges by investigating whether provision of a multi-component breastfeeding support intervention can improve the six-month EBF rate, as compared to current standard care. The intervention combines antenatal breastfeeding education with peer and professional lactation support, and is delivered in the hospital and home settings. To our knowledge, only one previous small study examined the combined effect of the three components showing a modest, albeit insignificant effect on breastfeeding outcomes [5,7].

## Materials and methods

This was a randomized, parallel-group, clinical trial, conducted between December 2013 and January 2016 in the obstetrics clinics of two academic tertiary care centers in Beirut, Lebanon. Trained research assistants reviewed the schedule of appointments of the prenatal clinics at both participating sites on daily basis, and identified eligible women. Eligible women were then directly approached for enrolment in the trial upon presentation to the clinic, and inclusion and exclusion criteria were validated by asking the pregnant women about each criterion. Inclusion criteria were healthy pregnancy in the first or second trimester, as determined by date of the last menstrual period, and intention to attempt breastfeeding after delivery. Exclusion criteria were pregnancy beyond the second trimester, maternal chronic medical condition such as hypertension or diabetes, abnormal fetal screen at 20–22 weeks, determined not to breastfeed, twin pregnancy, not residing in Lebanon for at least six months after delivery, or delivery before 37 weeks of gestation.

### Randomization and masking

Eligible women were randomly allocated, in a 1:1 ratio, to standard obstetric and pediatric care (control), or to a multi-component breastfeeding support intervention composed of antenatal education, peer, and professional lactation support (experimental). The allocation was computer-generated by one of the co-investigators (HT) who was not involved in recruitment. Stratified block randomization was carried out with block sizes varying between four and eight, with the sample randomized at each site being proportional to the volume of patients seen at that site (ratio of 4:1). Allocation concealment was ensured by using a set of sequentially numbered opaque sealed envelopes specifying group allocation. Trained assistants recruited women after verifying inclusion and exclusion criteria, and obtained written consent before the allocation was revealed. The assistants assured the timely delivery of the intervention components to participants in the experimental group, and hence could not be blinded. The investigators were also not blinded but were neither involved in data collection, nor in outcome assessment.

### Procedures

Participants in the control group received standard prenatal and postnatal care. In Lebanon, standard prenatal care is provided by obstetricians only, and is mainly focused on obstetrical care. Information relating to breastfeeding is not currently part of prenatal care in any region of the country. Advice on infant feeding is provided by pediatric physicians and nurses or midwives, usually after delivery. Moreover, hospitals and maternities do not have lactation consultants on their staff.

In addition to standard care, participants in the experimental group received the following intervention components: a) prenatal breastfeeding education to address common community misconceptions about breastfeeding and improve maternal knowledge and expectations, b) postpartum professional lactation support to avoid, and/or overcome technical breastfeeding challenges that mothers experience, and improve maternal self-efficacy through empowerment, c) postpartum peer (lay) support to provide emotional support, and build maternal social capital. Our multi-component intervention was based on the Social Network and Social Support Theory that offers a framework of pathways through which social ties can influence health [16].

The multi-component intervention started with antenatal education at least a week after enrolment, followed by peer and professional support on the first day postpartum, and continued for six months. Details of the intervention are available in the trial’s published protocol [17]. Briefly, prenatal breastfeeding education was delivered in one session by a certified lactation consultant, and participants were given a booklet detailing breastfeeding benefits, expectations, positioning techniques, and hazards of artificial milk, as well as a video that addressed common community misconceptions about breastfeeding. Peer support was provided by women (support mothers) who had successfully breastfed at least one child for a minimum of two months, and had positive breastfeeding attitudes. After attending two half-day training sessions, support mothers (SMs) contacted participants in the experimental group according to a pre-specified schedule. Support occurred in an informal manner based on a minimum number of 10 scheduled calls or hospital/home visits, starting with the antenatal class, then at the sixth and nineth months of gestation, the expected week of delivery, the first day postpartum, 48 hours from hospital discharge, one, two, and four weeks postpartum, and monthly thereafter until six months postpartum. This schedule could be modified based on the needs of the breastfeeding mother. Peer support continued until the infant was six months of age, or until the breastfeeding mother stopped breastfeeding or withdrew from the clinical trial, whichever came first [17]. Professional lactation support was delivered by certified lactation experts who visited the participants on the first postpartum day in the hospital, and continued with home visits on days three, seven, and fifteen postpartum, and then monthly for six months, or until breastfeeding discontinuation or maternal request to stop, whichever came first. Lactation support was provided mainly as face-to-face, but could also happen via telephone, if so requested by the participant. Additional visits were permitted if requested by the mother, or judged to be necessary by the lactation expert [17]. Participants in the control group received standard prenatal and postnatal care as dictated by their obstetricians and pediatricians. Optional prenatal classes about labor, delivery, and breastfeeding are available at one site but not at the other one. However, certified lactation consultants are unavailable at both centers.

At enrolment, and directly after obtaining the written informed consent, baseline data were collected by trained assistants who administered a structured questionnaire to collect information on socio-demographics, as well as four breastfeeding questionnaires that were validated in Arabic. These were the Iowa Infant Feeding Attitude Scale (IIFAS-A) [18], the Infant Feeding Intention Scale (IFI-A) [19], the Infant Breastfeeding Knowledge questionnaire (BFK-A) [20], and the Breastfeeding Behavior Questionnaire (BBQ-A) [21]. The IIFAS-A score is between 17 and 85 points, with higher scores reflecting more positive attitudes towards breastfeeding. IFI-A score ranges between 0 (strong intention to not breastfeed) and 16 (strong intention to exclusively breastfeed for up to 6 months). The BFK-A score is between 4 and 16 points with higher scores indicating better knowledge, and the BBQ-A score ranges from 12 to 72 points, with lower scores representing more positive breastfeeding behaviors than higher scores. At six months, participants were re-administered the BFK-A, IIFAS-A and BBQ-A scales to assess the changes in maternal breastfeeding knowledge, attitude, and behavior. Maternal satisfaction with her psychological status, infant’s health, socioeconomic status, relationship with spouse or partner, relations with family and friends, and health and functioning was assessed at one, three, and six months postpartum, using a questionnaire that was adapted from the Postpartum Quality of Life-Part A questionnaire [22]. This instrument has thirty six items that assess the extent of the respondent’s satisfaction with a specific situation using a six-point Likert scale ranging from *Very dissatisfied* to *Very satisfied*. At six months, the experimental group was surveyed about their satisfaction with peer and professional lactation support using a six-item, locally developed questionnaire. Each item assesses the extent of maternal agreement to a specific situation using a five-point Likert scale that ranges from *Strongly disagree* to *Strongly agree*.

### Outcome measures

Our primary endpoint was EBF rate at six months. EBF was defined according to the WHO as feeding the baby mother’s milk only, with no other food or drink including water, but allowing oral rehydrating solutions, vitamins, minerals or other medicines when needed [23]. Secondary endpoints were the differences in EBF rates at one and three months, rates of any breastfeeding (exclusive or mixed) at one, three, and six months, changes from baseline in maternal breastfeeding knowledge, behavior, and attitude at six months, maternal satisfaction with quality of life issues at one, three, and six months, satisfaction of participants in the experimental group with peer, and professional support at six months, and adverse events.

### Patient and public involvement

Pregnant or nursing women were not involved in setting the research question, study design and implementation, or the outcome measures. The findings of this trial were disseminated to the study participants in a conference.

### Statistical analysis

The six-month EBF rate in the control group was estimated at 2% based on the most recently reported EBF rate from Lebanon, at the time the protocol was developed [10]. We hypothesized that 12% of the experimental group will be exclusively breastfeeding at six months. To detect this 10% difference in the EBF rates of both groups, with 90% power, and 5% type I error, 155 mothers were needed per group. We inflated the sample size from 310 to 443 to accommodate for a potential 30% attrition rate.

Descriptive statistics summarized categorical variables as counts and proportions, and continuous variables as means and standard deviations, or as medians and interquartile ranges. The association between the intervention and categorical outcome variables was assessed using the Chi squared test, whereas the Student’s t-test was used for continuous outcomes. Moreover, multivariable stepwise logistic regression analysis was used to adjust for residual confounding for categorical outcomes, with *p*-value for entry in the model set at <0.2. We also carried out repeated measurement analyses using the generalized estimating equations (GEE) to explore the effect of the intervention over time. Statistical analyses were carried out based on the intention to treat principle.

Since some participants in the experimental group declined one or more components of the intervention, we conducted a post-hoc multivariable stepwise logistic regression analysis to explore whether the dose of the intervention received (number of components) would affect the rate of EBF at six months. In this analysis, we grouped participants who declined all three components with participants who received one component only. We also conducted a sensitivity post-hoc analysis in which participants that declined all three intervention components were treated as control. Results were reported as adjusted odds ratios and their 95% confidence intervals.

The Statistical Package for Social Sciences (SPSS) version 23 was used for data management and analyses. A *p*-value of <0·05 indicates statistical significance. This study was approved by the Institutional Review Boards of both sites, and is registered in Current Controlled Trials ISRCTN17875591. www.isrctn.com

## Results

**Fig 1:**
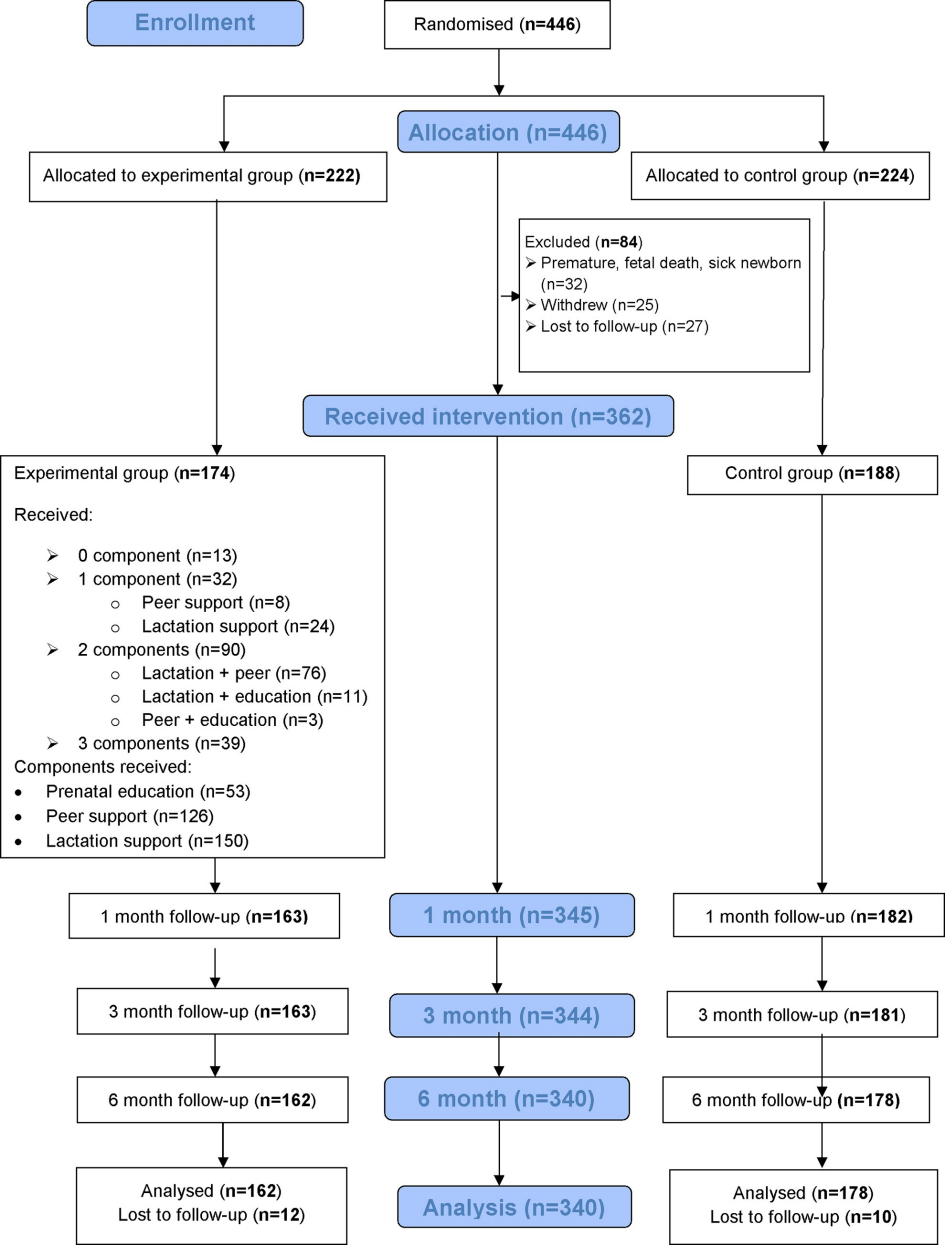
Participants’ flow through the trial.

Between December 2013 and January 2016, 446 women were enrolled and assigned to the control (n = 224), or to the experimental (n = 222) group. Of 446 participants, 362 (81.2%) received the allocated intervention, and 340 (93.9%) provided outcome assessment at six months. Reasons for drop out from the study are detailed in the flow diagram (Fig 1).

Table 1 summarizes the baseline characteristics of the two groups. Participants in the experimental group had higher monthly income, fewer children, shorter duration of previous breastfeeding, and fewer children who were breastfed (*p* ≤ 0.001). Moreover, a higher proportion of women in the experimental group had female pediatricians (*p* = 0.01). The remaining characteristics were similarly distributed between the two groups.

**Table 1:** Baseline characteristics.

Variable	Control (n = 188)	Intervention (n = 174)
**Site**		
Centre A	155 (82.4%)	155 (89.1%)
Centre B	33 (17.6%)	19 (0.1%)
**Age** (years) (mean ± SD)	29.27 ± 5.19	29.9 ± 4·53
**Employment**		
No	96 (51.1%)	80 (46.0%)
Yes	92 (48.9%)	94 (54.0%)
**Education**		
Intermediate and less	24 (12.8%)	13 (7.5%)
Secondary or technical	23 (12.2%)	16 (9.2%)
University	141 (75.0%)	145 (83.3%)
**Monthly income** (USD)*		
<1000	62 (34.6%)	31 (19.3%)
≥1000	117 (65.4%)	130 (80.7%)
**Gestational weeks at delivery** (mean ± SD)	38.55 ± 1.32	38.57 ± 1.32
**Mode of delivery**		
Vaginal	106 (58.2%)	103 (64.0%)
Caesarean-section	76 (41.8%)	58 (36.0%)
**Has support at home**	182 (96.8%)	171 (98.3%)
**Male newborn gender**	102 (56.0%)	89 (55.3%)
**IFI-A score** (mean ± SD)	12.26 ± 3.01	12.61 ± 2.89
**BFK-A score** (mean ± SD)	11.41 ± 2.49	11.04 ± 2.54
**IIFAS-A score** (mean ± SD)	65.88 ± 7.28	65.35 ± 7.15
**BBQ-A score**		
Positive	97 (51.6%)	93 (53.8%)
Negative	91 (48.4%)	80 (46.2%)
Number of children* Median (IQR)	1.0 (0.0–2.0)	0.0 (0.0–1.0)
Number of breastfed children* Median (IQR)	0.0 (1.0–1.8)	0.0 (0.0–1.0)
Duration of previous breastfeeding (months)* (mean ± SD)	11.86 ± 8.25	7.82 ± 6.02
**Male paediatrician** #	113 (63.5%)	78 (48.8%)
**Practiced rooming in**	81 (45.5%)	89 (55.6%)
**Breast milk as baby’s first feed**	134 (75.3%)	121 (75.2%)

*p ≤ 0.001

#*p* = 0.01; IQR: Interquartile range.

### Breastfeeding outcomes

At six months, 57 of 162 (35.2%) participants in the experimental group were exclusively breastfeeding, as compared to 50 of 178 (28.1%) in the control group (*p* = 0.16). There were no significant differences in the crude EBF rates at one and three months, nor in the rates of any breastfeeding at one, three, and six months (Table 2). Moreover, the crude association between group allocation and EBF at six months was insignificant (OR = 1.39; *95%CI*: 0.88 to 2.20).

**Table 2:** Crude analysis of breastfeeding outcomes at 1, 3 and 6 months.

Time	Outcome	Control n (%)	Intervention n (%)	*p*-value
**1 month**	EBF Not EBF	91 (50.0%)	87 (53.0%)	0.53
		91 (50.0%)	76 (46.6%)	
	Any BF Artificial milk	172 (94.5%)	153 (95.0%)	0.83
		10 (5.5%)	8 (5.0%)	
**3 months**	EBF Not EBF	73 (40.3%)	69 (42.3%)	0.70
		108 (59.7%)	94 (57.7%)	
	Any BF Artificial milk	137 (78.7%)	121 (76.6%)	0.64
		37 (21.3%)	37 (23.4%)	
**6 months**	EBF Not EBF	50 (28.1%)	57 (35.2%)	0.16
		128 (71.9%)	105 (64.8%)	
	Any BF Artificial milk	101 (61.6%)	88 (59.1%)	0.65
		63 (38.4%)	61 (40.9%)	

EBF: exclusive breastfeeding; BF: breastfeeding.

In the multivariable stepwise logistic regression model that adjusted for site, monthly income, gender of pediatrician, rooming in, breastfeeding behavior category, duration of previous breastfeeding, number of children, number of breastfed children, and group allocation, participants in the experimental group were twice as likely to continue EBF for six months. Other positive predictors of EBF at six months were history of a longer duration of previous breastfeeding, and having fewer children (Table 3).

**Table 3:** Predictors of six-month exclusive breastfeeding in the multivariable stepwise regression model.

Predictors	OR (*95% CI*)	*p*-value
**Multi-component intervention**	2.02 (1.20 to 3.39)	0.008
**Previous BF duration**	1.12 (1.07 to 1.17)	<0.001
**Number of children**	0.63 (0.44 to 0.90)	0.011

BF: breastfeeding. Variables in the model: site, monthly income, gender of pediatrician, rooming in, breastfeeding behaviour category, duration of previous breastfeeding, number of children, number of breastfed children, and group allocation.

Moreover, the GEE analysis revealed a significant effect of the intervention on EBF (*p* = 0.016), but the effect of the intervention over time was not found to be significantly different (*p* = 0.621). Among participants in the experimental group (n = 174), only 39 (22.4%) complied with all three intervention components, whereas 90 (51.7%) complied with two components, 32 (18.4%) complied with one component, and 13 (7.5%) did not comply with any of the components (Fig 1). The post-hoc multivariable logistic regression analysis suggested a dose-response effect of the multi-component intervention. Women receiving all three components were 6.6 times more likely to practice EBF for six months as compared to controls (OR = 6.63; 9*5%CI*: 3.03 to 14.51). Participants receiving two components were 1.7 times more likely than controls to be exclusively breastfeeding at six months, but this was not statistically significant. Similar results were obtained in the post-hoc sensitivity analysis in which participants declining all three intervention components were treated as controls (Table 4).

**Table 4:** Post-hoc multivariable stepwise regression analysis of six-month breastfeeding outcomes in the intervention group by the number of intervention components received.

	Predictors	OR (*95% CI*)	*p*-value
**Model 1**	One intervention component	0.51 (0.17 to 1.56)	0.235
	Two intervention components	1.78 (0.97 to 3.26)	0.062
	Three intervention components	6.63 (3.03 to 14.51)	<0.001
	Previous BF duration	1.12 (1.07 to 1.17)	<0.001
	Number of children	0.66 (0.46 to 0.98)	0.028
**Model 2**	One intervention component	0.71 (0.23 to 2.22)	0.553
	Two intervention components	1.89 (1.03 to 3.45)	0.039
	Three intervention components	7.08 (3.24 to 15.46)	<0.001
	Previous BF duration	1.13 (1.08 to 1.18)	<0.001
	Number of children	0.66 (0.46 to 0.95)	0.027

Model 1: Participants who declined all three intervention components were treated as having received one component; Model 2: Participants who declined declining all three intervention components were treated as controls.

BF: breastfeeding. Variables in the model = site, monthly income, gender of pediatrician, rooming in, breastfeeding behaviour category, duration of previous breastfeeding, number of children, number of breastfed children, and group allocation.

### Breastfeeding questionnaires

The two groups had significantly higher BFK-A and IIFAS-A scores at six months, as compared to baseline, which translates to better breastfeeding knowledge, and more positive attitude. Whereas six-month BFK-A scores were much higher in the experimental group, there were no differences in the IIFAS-A scores of both groups indicating similar breastfeeding attitudes in both groups towards the end of the trial. Six-month BBQ-A scores were slightly lower than baseline values in both groups, indicating more positive breastfeeding behaviour. However, this change in BBQ-A scores from baseline was significant in the experimental group only (Table 5).

**Table 5:** Participants’ scores on breastfeeding questionnaires at baseline, and at six months.

Questionnaire	Control		Intervention		Between Group Comparison of Mean Difference Between Baseline and 6 Months
	Mean (SD)	P value	Mean (SD)	P value	P value
**BFK-A**					
Baseline	11.4 (2.5)	0.003	11.2 (2.5)	<0.001	<0.001
6 months	12.0 (2.5)		13.1 (2.1)		
**IIFAS-A**		0.003		<0.001	0.077
Baseline	65.9 (7.4)		65.7 (7.2)		
6 months	67.6 (8.0)		69.1 (7.8)		
**BBQ-A**		0.210		0.014	0.347
Baseline	28.0 (8.0)		28.3 (7.3)		
6 months	27.1 (8.1)		26.4 (8.1)		

In stratified analysis by breastfeeding status at six months, women who continued EBF for six months had better breastfeeding knowledge, stronger breastfeeding attitudes, and more positive breastfeeding behavior at six-months, irrespective of their group allocation (Table 6). Moreover, participants who continued EBF for six months had significantly higher baseline IFI-A scores (mean difference = 1.52; *95%CI*: 0.85 to 2·19), irrespective of their group allocation. This difference in baseline IFI-A scores between those who continued six months of EBF, and those who stopped earlier was more pronounced in the control group (mean difference = 1.77; *95%CI*: 0.79 to 2.76), as compared to the experimental group (mean difference = 1.21; *95%CI*: 0.29 to 2.13).

**Table 6:** Infant feeding type and six-month scores on BFK-A, IIFAS-A and BBQ-A.

Infant feeding type	IIFAS-A		BFK-A		BBQ-A	
	Score	*P* Value	Score	*P* Value	Score	*P* Value
**Overall**		<0.001		<0.001		<0.001
EBF	71.8 (5.8)		13.5 (2.1)		24.0 (7.2)	
Any BF	66.4 (8.4)		11.9 (2.4)		28.1 (8.1)	
**Intervention**		<0.001		<0.001		<0.001
EBF	72.6 (5.1)		13.9 (1.7)		24.3 (7.3)	
Any BF	66.7 (8.5)		12.5 (2.1)		27.8 (8.3)	
**Control**		<0.001		<0.001		<0.001
EBF	70.9 (6.4)		13.0 (2.3)		23.7 (7.2)	
Any BF	66.1 (8.4)		11.5 (2.5)		28.5 (8.0)	

IIFAS-A: Iowa Infant Feeding Attitude Scale-Arabic; BFK-A: Infant Breastfeeding Knowledge questionnaire-Arabic; BBQ-A: Breastfeeding Behaviour Questionnaire-Arabic; EBF: Exclusive breastfeeding; BF: Breastfeeding.

### Satisfaction outcomes

Maternal satisfaction with quality of life issues was similar in both groups at one, three and six months (Table 7).

**Table 7:** Maternal satisfaction with quality of life issues.

Time	Group	Mean	SD	P value
**First month**	Control	194.1	21.6	0.687
	Intervention	193.1	23.3	
**Third month**	Control	195.7	21.5	0.667
	Intervention	194.6	24.1	
**Sixth month**	Control	194.3	21.8	0.784
	Intervention	193.6	24.0	

The majority of participants in the experimental group were satisfied with their experience with peer and professional lactation support (Table 8). There were no adverse events reported by any of the participants during the conduct of the study.

**Table 8:** Maternal satisfaction with peer and professional support.

Question	Agree n (%)	Neutral n (%)	Disagree n (%)
I had a positive experience with:			
- my SM	83 (86.5)	9 (9.4)	4 (4.2)
- my LC	126 (95.5)	3 (2.3)	3 (2.3)
I felt pressured to continue BF from:			
- my SM	1 (1.1)	4 (4.2)	90 (94.7)
- my LC	0 (6.1)	3 (2.3)	121 (91.7)
I felt judged when I did not succeed/comply with:			
- my SM’s suggestions	0 (0.0)	2 (2.1)	93 (97.9)
- my LC's suggestions	2 (1.5)	3 (2.3)	127 (96.2)
If I could do it over again, I would:			
- have a SM	80 (84.2)	5 (5.3)	10 (10.5)
- have a LC	116 (87.9)	6 (4.5)	10 (7.6)
I had enough contact with:			
- my SM to help me with BF	64 (67.4)	15 (15.8)	16 (16.8)
- my LC to help me with BF	121 (91.7)	7 (5.3)	4 (3.0)
My contact with:			
- my SM positively affected the duration of my BF	63 (66.3)	25 (26.3)	7 (7.4)
- my LC positively affected the duration of my BF	119 (90.2)	10 (7.6)	3 (2.3)
Given the opportunity, I would consider becoming a SM myself	73 (77.7)	4 (4.3)	17 (18.1)

SM: Support mother; LC: Lactation consultant; BF: Breastfeeding.

## Discussion

This study builds on the existing literature, and provides further evidence on the additive positive effect of combining antenatal breastfeeding education with peer and professional lactation support on exclusive breastfeeding, when delivered as a continuum that starts in the hospital, and extends to the home setting. The multi-component intervention doubled six-month EBF as opposed to standard of care. There was a positive association between the number of intervention components received by the participants in the experimental group and EBF at six months. Moreover, the combined intervention improved BF knowledge of the intervention group significantly more than that of the control group, but did not affect their attitude or behaviour. The improvement in breastfeeding knowledge noted in the control group could be attributed to the “placebo effect”. The lack of effect on attitude and behavior may be explained by the fact that intent to breastfeed was one of the trial’s inclusion criteria. Hence the intervention may fail to have a significant impact on the attitude or behavior of an already motivated mother. This is further supported by the relatively high six-month EBF rate in the control group (28.1%), which is much higher than what has been previously reported from Lebanon (2%-15%) [10,24].

Our findings are in agreement with a previous systematic review, in which the combination of education, lay, and professional support had a modest effect on EBF at four to six months (RR = 2.07; *95%CI*: 0.98 to 4.39) [5]. However, the study that contributed to this outcome was small with unclear allocation concealment, blinding, and selective reporting bias, and suffered from incomplete outcome data assessment [5,7]. In contrast, our trial had adequate allocation concealment, a large sample size, with 94% of the randomized cohort providing information on the primary outcome at six months. We analyzed and reported on all outcomes as prespecified in the published protocol [17]. Our findings also agree with previous studies reporting improved exclusive breastfeeding initiation and continuation, when interventions were delivered in both health systems and home settings [8], or during both prenatal and postnatal periods [25].

There are some limitations to our study. All participants came from an urban setting, and had to have the intention to breastfeed as an inclusion criterion. Hence, the findings may not be generalizable to women who are determined not to breastfeed for various reasons, or to women who are in rural settings, as their acceptance of peer or professional support may be different. Another limitation concerns the open-label nature of the trial. Although the trial was initially planned as participant-blind, yet because of the nature of the intervention components (specifically peer support and professional lactation support), it is possible for participants in the experimental group who have previous children to guess that they were allocated to the active arm. Hence, there is potential for reporting bias. However, this bias is minimized by the fact that the breastfeeding outcomes of participants in the experimental group who received professional lactation support (150 of 174 women) were verified by the lactation consultants during their home visits to the mothers. Blinding of the outcome assessors was also not feasible, since they were directly responsible for assuring the timeliness and quality of peer and professional support provision. For the control group, lack of blinding of outcome assessors is unlikely to cause significant detection bias, since the primary outcome of six-month EBF was reported by the control participants. In the experimental group, the risk for detection bias is minimized by the fact that breastfeeding outcomes were verified by the lactation consultants, except in participants who declined this component (24 of 174 women). Another potential limitation is that contamination between the two groups could happen given the nature of the multi-component intervention. However, our outcome assessors did not identify any such cases during their contact with the participants. A fourth limitation is the fact that only 39 of the 174 participants in the experimental group complied with all 3 intervention components. Such non-compliance compromises the causality association between the multi-component intervention and the sixth-month EBF. Hence, the finding of a dose-response association between the number of intervention components and six-month EBF should be interpreted with caution, since it is generated from post-hoc analysis.

In conclusion, our study highlights the additive effect of multiple interventions delivered in health care and home settings in supporting breastfeeding mothers, and bolstering exclusive breastfeeding practice in the community, by utilizing the social network and professional lactation experts. A cost-effectiveness study of this strategy is needed at the national level. Previous evidence reveals that breastfeeding support interventions are cost-effective [26], and that the cost of not breastfeeding consumes about 0.49% of the world’s gross domestic product [27]. Further studies are also needed to explore how best to address women who have negative attitudes towards breastfeeding.

## Supporting information

S1 Checklist Consort checklist. (DOC)

S1 Dataset SPSS dataset. (SAV)

S1 Protocol Study protocol. (PDF)
