# Supplementary material for: A multi-component intervention to support breastfeeding in Lebanon: A randomized clinical trial
Source: PLoS One. 2019 Jun 14;14(6):e0218467. doi: 10.1371/journal.pone.0218467 (PMC6568407; doi:10.1371/journal.pone.0218467)
Supplement: S1 Protocol — (PDF) [file pone.0218467.s003.pdf]

A complex breastfeeding promotion and support intervention in a developing country: a randomized clinical trial

Research Proposal

Mona Nabulsi, MD, MS and Haya Hamadeh, MD, MPH
November-2012

PRINCIPAL INVESTIGATORS:

Name

Mona Nabulsi, MD, MS

Haya Hamadeh, MD, MPH

FACULTY: Faculty of Medicine

DEPARTMENT: Pediatrics and Adolescent Medicine

DATE OF SUBMISSION: November 2012

[**X**] New Proposal [ ] Renewal

SUBMITTED TO: Allen Foundation Inc.

STARTING DATE OF STUDY: October 1, 2013

DATE OF STUDY COMPLETION: October 1, 2018

COLLABORATORS:

**Name**	**Affiliation**
Tamar Kabakian, PhD	Faculty of Health Sciences
Hani Tamim, PhD	Biostatistics
Lama Charafeddine, MD	Pediatrics & Adolescent Medicine
Durriyah Sinno, MD	Pediatrics & Adolescent Medicine
Saadieh Sidani, BSN	School of Nursing
Nadine Yehya, PhD	Olayan School of Business

Abstract

Breastfeeding has countless benefits to mothers, children and community at large, especially in developing countries. Studies from Lebanon report disappointingly low breastfeeding exclusivity and continuation rates. Exclusive breastfeeding is a cost-effective public health measure that has a significant impact on infant morbidity and mortality. In a country with limited healthcare resources like Lebanon, developing an effective breastfeeding promotion and support intervention that is easily replicated across various settings becomes a priority. Evidence reveals that antenatal breastfeeding education, professional lactation support, and peer lay support are individually effective at increasing breastfeeding duration and exclusivity, particularly in low-income settings. Given the complex nature of the breastfeeding ecosystem and its barriers in Lebanon, we hypothesize that a complex breastfeeding support intervention, which is centered on the three components mentioned above, would significantly increase breastfeeding rates. We plan on conducting a multi-center randomized controlled trial with 443 participants who will be followed from early pregnancy to five years after delivery. The intervention will consist of a "prenatal/postnatal" professional and peer breastfeeding support package, guided by the Social Network and Social Support Theory. We will use a quantitative methodology to assess breastfeeding exclusivity and continuity rates at 1, 3 and 6 months. We also plan to conduct an assessment of mothers' postpartum quality of life in addition to their breastfeeding knowledge, attitudes and behavior. Finally, a cost-benefit assessment of the intervention will be determined for advocacy and national dissemination purposes. To our knowledge, this is the first study of its kind to be conducted in the MENA region. If positive, the results would provide a generalizable model to bolster breastfeeding promotion efforts and contribute to improved child health.

Background

Despite its countless benefits to children, mothers and community at large, breastfeeding rates in Lebanon continue to be disappointingly low. Previous studies reported acceptable initiation rates varying between 63.8% and 96% [1, 2]. However, exclusive breastfeeding is reported in 58.3% of babies less than one month, and in 10.1% to 4.1% of 6-month old infants [3-6]. Only 27.1% of one-year old infants continue to breastfeed [3].

Breastfeeding is associated with reduced infant risks of infections, atopic dermatitis, asthma, obesity, diabetes types 1 and 2, childhood leukemia, sudden infant death syndrome, necrotizing enterocolitis; and with higher Intelligence Quotient and academic performance at 6.5 years of age [7-9]. Moreover, it is associated with decreased maternal risks of diabetes type 2, breast and ovarian cancers, and postpartum depression [7]. As such, breastfeeding is a cost-effective public health measure that has a significant impact on infant morbidity and mortality in developing countries [10, 11]. Exclusive breastfeeding for 6 months and continued until 11 months of age was the single most effective strategy to improve child survival in developing countries, preventing 13% of under-five mortality [12].

Several cross-sectional studies from Lebanon reported different predictors of low breastfeeding rates. These included lower socio-economic status, Caesarean birth, urban residence, early hospital discharge, mother's religion, male paediatrician, and hospital practices that hinder breastfeeding like lack of rooming in of mother and baby, fixed newborn feeding schedules, and offering glucose and water or artificial formula as first feeds instead of breast milk [2, 4, 13]. A recent qualitative study that explored breastfeeding perceptions and experiences of 36 mothers who were followed up longitudinally for one year reported several barriers to breastfeeding exclusivity and continuation in the Lebanese context [14]. Barriers included several maternal and community misconceptions such as insufficiency of breast milk and lack of satiety in the baby, breastfeeding causing maternal weight gain or breast sagging, harmful maternal milk during certain situations such as grief, maternal illness or pregnancy. Moreover, mothers complained of breastfeeding causing pain, sleep deprivation, and exhaustion. On the other hand, women who continued breastfeeding for one year were cognizant of the difficulties of breastfeeding and showed determination to succeed and overcome any barrier, relying mostly on family support and proper time management. That study uncovered the need for several interventions that can address the different barriers in the Lebanese context and the need to empower mothers to overcome them, hoping to improve the existing national breastfeeding exclusivity and continuation rates.

Of the different interventions reported in the literature to improve breastfeeding rates, breastfeeding support, whether professional or lay, was quite effective in increasing breastfeeding duration [15-18]. In particular, lay support with or without a professional component increased the rate of any short- or long-term breastfeeding, as well as the rate of short-term exclusive breastfeeding duration [16]. Lay breastfeeding support also has additional health benefits in several aspects of families' lives such as playing a role in reducing obesity and postpartum depression [17]. Evidence suggests it tends to be more effective in low- and middle-income settings [18]. Similarly, peer support interventions had a significantly greater effect on any breastfeeding in low- or middle-income

countries, reducing the risk of not breastfeeding at all by 30%, compared with a reduction of 7% in high-income countries. Moreover, the risk of non-exclusive breastfeeding decreased significantly more in low- or middle-income countries than in high-income countries with these interventions [18]. A recent review of four randomized controlled trials, including one from Syria showed that community-based interventions were significantly associated with an increase in exclusive breastfeeding rates at four and six months after [19]. Interestingly, a mother-to-mother breastfeeding line that was established in Toronto and Nova Scotia to promote and support breastfeeding women succeeded in achieving one hundred percent breastfeeding rate by the end of the third month among the women who participated in The Yarmouth Friendly Feeding Line (YFFL) pilot [20]. Also, a recent Cochrane review that assessed the effectiveness of support for breastfeeding mothers of healthy term babies found that all forms of extra support analyzed together increased the duration of exclusive breastfeeding at 6 months (RR=0.86; 95%CI: 0.82 to 0.91). Support was more effective in settings with high initiation rates (like Lebanon); with strategies that rely on face-to-face support being more likely to succeed as opposed to support that is offered reactively upon mothers' request [21].

Breastfeeding education is another intervention that was associated with a significant increase in initiation rates, specifically in low-income USA women as compared to standard of care (RR 1.57, 95%CI: 1.15 to 2.15) [22]. In particular, one-to-one, needs-based, informal repeat sessions, and generic formal antenatal education were effective in increasing breastfeeding rates. Needs-based, informal *peer* support, whether ante-natal or post-natal were particularly effective in increasing initiation rates (RR 4.02, 95%CI: 2.63 to 6.14) [22].

The totality of evidence underscores the heterogeneous and complex nature of breastfeeding barriers in any given setting. Evidence thus suggests that in order to effectively raise breastfeeding rates, there is need for multi-dimensional interventions that simultaneously tackle different aspects of breastfeeding. In this proposed randomized clinical trial, we plan to investigate whether a complex breastfeeding promotion and support intervention in early pregnancy is effective in improving six-month breastfeeding exclusivity rates. We hypothesize that a complex intervention composed of several simple interventions that were previously shown to individually improve breastfeeding rates in low- and middle-income countries may be more effective than each individual intervention alone in improving breastfeeding exclusivity rate in Lebanon, one of the lowest in the region [23].

Our proposed complex intervention is based on the Social Network and Social Support theory framework, which is a theory on the interpersonal level that offers a framework describing pathways through which social ties can influence health [24]. Social network denotes to the web of social ties or relationships of a person through which this person receives social support. Social networks are characterized by the dyadic characteristics of 1) reciprocity, the extent to which support is given and received in a relationship, 2) intensity, the emotional closeness experienced in a relationship, and, 3) complexity, the variety of functions that the relationship serves. In addition, the characteristics of homogeneity in terms of demographic characteristics, geographical dispersion in terms of

proximity to the focal person and density in terms of the extent of interaction of members, are used to describe the network as a whole. Social networks represent relationships between people that provide social support as one of their functions. Social support is categorized in four supportive behaviors:

1. Emotional support: conveying that a person is valued to be cared for in ways that is health promoting.
2. Instrumental support: provision of aid and services.
3. Appraisal: provision of information for self-evaluation; constructive feedback.
4. Sharing points of view: offering opinions about how one would handle a situation.
5. Informational support: provision of advice or information to address a particular situation.

Hence, our complex intervention consists of all the following simpler interventions: 1) breastfeeding education and counseling to improve knowledge and awareness of expectations; 2) building of appropriate breastfeeding skills to improve self-efficacy and empower breastfeeding mothers; 3) providing professional lactation support; 4) establishing mother-to mother tree of lay support. This intervention will develop new social network linkages and will use members of women's own social networks to enhance their role in breastfeeding support.

To our knowledge, this is the first study of its kind to be conducted in the Lebanon and the region as no previous studies investigated the effectiveness of a complex intervention that is composed of several interventions previously shown to improve breastfeeding rates in low- or middle-income countries. Should this intervention prove to be effective, it can be used as a framework for a model intervention that may be replicated in any setting or community in Lebanon, whether rural or urban.

Objective

To investigate whether a complex intervention targeting new mothers' breastfeeding knowledge, skills and social support within a Social Network and Social Support theory framework will increase exclusive breastfeeding duration among women in Lebanon.

Specific Aims

To investigate the effect of the complex intervention on the following outcomes in the intervention group, in comparison to the control group:

1. Maternal breastfeeding skills (following professional lactation support in the hospital and later at the home).
2. Maternal knowledge of breastfeeding benefits, and awareness of formula hazards and breastfeeding difficulties (following formal antenatal education)
3. Maternal self-efficacy (following the establishment of a 24-hour professional and lay support system using a breastfeeding hotline, peer support network, and family engagement).
4. Quality of life.

5. Economic efficiency.

Research Design and Methods

This is a randomized controlled single-blind clinical trial that will be conducted in two centers: the Women's Health Center of the American University of Beirut Medical Center (AUBMC), and the Obstetrics Clinics of Sahel General Hospital (SGH), Beirut-Lebanon. Healthy pregnant women who are in their first trimester and who intend to breastfeed after delivery will be approached for enrolment in the study. Women with any of the following conditions will be excluded: pregnancy beyond the first trimester, chronic medical condition, abnormal fetal screen (ultrasound/blood/amniocentesis), not willing to breastfeed later, not living in Lebanon for at least six months after delivery, twin gestation, and preterm birth (at <37 weeks gestation). Eligible pregnant women will be randomly allocated to one of two parallel groups (experimental and control), using a computer-generated stratified block randomization that is done by one of the co-investigators (HT) who is not involved in subject recruitment. The size of blocks will vary from 4-8 and the stratification will be by study site. The generated random allocation list will be concealed from the investigators and recruiters and will be kept with HT until the end of the trial.

Women in the experimental group will receive a complex intervention composed of the following elements: a) prenatal breastfeeding education to raise knowledge and awareness, b) postpartum professional lactation support to improve maternal skills and self-efficacy, c) postpartum peer (lay) support to build social support, and enhance social capital within women's social network. These include skill building activities for the provision of effective breastfeeding support.

Subjects in the control group will receive standard prenatal and postnatal care that is usually offered to mothers at both sites. At AUBMC, standard prenatal care includes prenatal classes that cover issues related to labor and delivery and do not go over breastfeeding. Women who wish to attend a prenatal session will have to self-register. After delivery, mothers are usually instructed on breastfeeding by the nurses and their pediatricians. At SGH, there are no structured prenatal classes and any education or training on breastfeeding is often done by the pediatricians at the hospital or later during well-baby checkups.

Components of the complex intervention

1. Prenatal breastfeeding education:
    a. *Antenatal classes:* Upon enrolment and signing of the written informed consent, each subject in the intervention group will be invited to attend at least one antenatal session with as many members of her family as she wishes. Those sessions are meant to be a forum to discuss breastfeeding information and to address the family's questions. They will be on schedule basis and open to the intervention group only in order to avoid contamination with the control group. Details of the objectives, content,

format and description of the antenatal class appear in APPENDIX-A. Data on socio-demographic variables, baseline breastfeeding knowledge, behavior and attitude towards breastfeeding will be collected at the beginning of each session from each subject. We will use a "Breastfeeding Knowledge, Attitude, and Behavior" questionnaire (B-KAB) that will be developed and adapted from the following validated instruments: the Iowa Infant Feeding Attitude Scale (IIFAS) [25], the Infant Feeding Intention Scale (IFI) [26], the Breastfeeding Behavior Questionnaire (BBQ) [27, 28], and the Infant Feeding Knowledge Test [29]. Those instruments are included in APPENDIX-B. The B-KAB questionnaire will be developed through a discussion among all investigators for adjustments before generating the first version. After reaching a consensus, two experts in breastfeeding will be asked to review the questionnaire for content validity and cultural appropriateness. It will then be translated to formal Arabic (Grade 4 literacy level) by an expert translator. The Arabic questionnaire will be back translated to English by another independent translator. The two translations will be checked independently by two bilingual investigators for accuracy of translation. Following content validity, field testing will be conducted on 20 women to inquire about the questionnaire's clarity and ease of comprehension. Any concerns, comments or suggestions will be noted, and necessary changes will be made before generating the final Arabic B-KAB questionnaire. Test-retest reliability will be established using a group of 20 students. Pearson correlation coefficient > 0.7 will be considered acceptable.

b. *Breastfeeding pamphlet and Video:* At the end of each antenatal class, subjects in the intervention group will get an educational pamphlet and video that contains information on breastfeeding benefits, techniques and expectations, as well as information on formula hazards (APPENDIX-C) and will be instructed to share them later with their family members (husband, mother, and mother-in-law).

2. Professional lactation support:

   In order to increase their breastfeeding skills and self-efficacy, mothers in the intervention group will be visited on daily basis during their hospital stay by a trained pediatric nurse (professional lactation support). Visits will be 15-30 minutes long and will entail hands-on training on breastfeeding positioning and latch as well as breast care and common breastfeeding concerns. To ensure breastfeeding continuity after hospital discharge, mothers will be visited in their homes on days 1, 3, 7 and 15, and then monthly until the 6th month postpartum, breastfeeding discontinuation, or until the mother requests to stop, whichever occurs earlier. Each nurse will be provided with a telephone to provide a 24-hour hotline service for additional breastfeeding support. The nurses will answer questions relating to breastfeeding and refer to a physician when appropriate.

3. Social network and social support:

To enrich a breastfeeding mother's social network and strengthen her social support, we will establish mother-to-mother networks intended to provide lay peer support. Support mothers (SM) will be recruited to provide breastfeeding mothers in the intervention group (BFMi) with peer support. Two different methods will be used to recruit SMs:

a) Enhancing existing social network linkages: Each enrolled participant will be asked to identify 1 – 3 women in her community or family whom she believes can serve as a source of support to her efforts to breastfeed successfully. Each BFMi will be asked to contact potential SMs and request they contact study recruiters for possible enrollment. Recruiters will receive potential SM calls and set a time and place to conduct an interview with the research team. During the interview, the mother-to-mother support program will be explained. Candidates possessing the necessary skills (APPENDIX-D) and willing to be support mothers will be enrolled.
b) Developing new social network linkages: Flyers will be posted on bulletin boards of AUBMC pediatric and obstetric clinics as well as SGH obstetric and pediatric clinics after their permission (APPENDIX-E). Eligible candidates enrolled in this manner will be matched with intervention subjects who were not able to identify possible SMs in their community and/or those whose nominated candidates were not considered suitable by our research team. The matching process will be based on age, availability and geographical distance. Similarly to above, clinic patients who are interested in becoming support mothers will be referred to the research team by their primary care physician.

Using these methods, we hope to enroll close to 74 SMs or approximately 1SM for every 2-3 BFMi. Breastfeeding support will occur in an informal manner based on a minimum number of scheduled calls/visits as follows:

Number	Type of contact	Date of contact
1	Face-to-face	First antenatal class
2	Telephone call	6th month of gestation
3	Telephone call	Beginning of 9th month
4	Telephone call	Week of expected delivery
5	Visit	First day postpartum
6	Visit	At 48 hours from discharge
7	Visit	After 1 week
8	Visit	After 2 weeks

9	Visit	After 4 weeks
10-14	Visit	Monthly till 6 months

Each BFMi will be given detailed instructions for contacting her SM when going to delivery suite or immediately after delivery. Employed mothers who need to go back to their jobs after 4-6 weeks from delivery may be visited a week before the end of their maternity leave if they wish so. The monthly visits will stop after six months, or earlier if the BFMi stops breastfeeding prior to 6 months. This schedule can be modified based on the needs of the BFMi. After each visit or call, support mothers will fill the support mother activity record (see APPENDIX-F). Support continues until the baby is 6 months of age or until the breastfeeding mother decides to stop, whichever comes first.

Training details

1. Professional lactation nurses: The two pediatric nurses will undergo extensive training on breastfeeding by taking the 20-hour course on breastfeeding developed by WHO/UNICEF. They will also sit for the International lactation consultant exam to become certified. Once the trial is over, the nurses will help deliver structured training in breastfeeding for AUBMC nurses who work in normal nursery, NICU, delivery suite, maternity ward, and obstetric clinics, as envisioned by the breastfeeding taskforce. As such, we believe that investment in those two nurses will be cost-effective on the long run to help transform AUBMC into a Baby-Friendly Hospital.
2. Recruiters: Two research assistants will be trained to approach and contact potential subjects at both AUBMC and SGH sites. Training will entail familiarizing them with all necessary documentation, including enrollment and consent forms in a structured 2-hour workshop. They will be trained to consent mothers in accordance with the ethical principles of informed consent, and call them using a prepaid phone card. Instructions and demonstrations on how to use these cards will also be given during the same workshop.
3. Support mothers: Training of SMs will take place at AUBMC at an agreed upon time and place. One of 3 pediatricians -part of the research team- will conduct seven training workshops (APPENDIX-G). Each workshop consists of two 2-hour sessions and will include a maximum of 10 SM participants. The first of the 2 sessions will be mainly theoretical. It will entail a brief overview of the mother-to-mother support program and training on the LOVE (listen observe validate empower/educate) method of support. In the second part of the first session, “breastfeeding basics” will be discussed, including advantages of breast milk and risks of formula; common culture-specific misconceptions will be addressed and proper technique of breastfeeding will be demonstrated using visual aid material. Breastfeeding trouble-shooting, and criteria for referral to appropriate medical services will also be emphasized. These criteria aim to identify mothers with medical conditions such as mastitis or breast abscess and postpartum depression

or psychosis as well as infant medical conditions, such as hyperbilirubinemia and/or dehydration. During the second session, which will be largely practical, support mothers will be asked to review what they have learned through interactive discussions; they will engage in role playing of “what if” scenarios as necessary, including failure to breastfeed scenarios. For those specific cases, SMs empathy and positive attitude will be emphasized. They will subsequently be handed a small manual containing a list of local breastfeeding resources as well as their telephone log sheets and activity record. At the end of each session a 10$ stipend will be given to each mother to cover transportation and/or parking cost. A healthy snack will be offered during each training session.

Process evaluation

During the support period, an ongoing evaluation process will take place in order to identify potential problems and implement changes accordingly. The process evaluation will ensure that the intervention is delivered and implemented as planned by evaluating the dose delivered, dose received, and reached.

The study research assistant will contact BFMis and SMs bi-weekly to inquire about the support process, including address potential complaints. The inquiry findings will be shared with the PI within one business day. Process amendments and/or improvements will take place within the same week if necessary. In case of a perceived need for referral by SMs, physician members of the research team will be notified within the same day and will contact the BFMi-SM pair in question to evaluate and confirm the medical urgency and refer-or not- accordingly. At the end of the support period, all records are returned to the research team. When the latter receives the records, a post-support survey is sent to the breastfeeding mother to fill and return for data entry and analysis (APPENDIX-H). Separate surveys will be sent to each support mother (APPENDIX-I) and to the professional nurses to assess their satisfaction (APPENDIX-J). All BFMis will be contacted by the research team on yearly basis for 5 years after the trial ends to ask about the success of exclusive breastfeeding of future babies. In addition, all mothers in the control group will be contacted by the research team at 1 month, 3 months and 6 months after delivery for data collection and outcome assessment similar to the intervention group. They will also be contacted on yearly basis for 5 years after the trial ends to ask about the success of exclusive breastfeeding of future babies.

Study endpoints

Our primary outcome will be the percent difference in 6-month breastfeeding exclusivity rates between the intervention and control groups.

Secondary outcomes include differences between the two groups with respect to the following:

a) Breastfeeding exclusivity rates at 1 month and 3 months.
b) Breastfeeding continuity rates at 1, 3 and 6 months.
c) Breastfeeding knowledge and attitudes of mothers at 6 months.

d) Success of mother-to-mother support system measured as satisfaction rates of BFMis, SMs, and nurses; BFMi referral rate and adverse events at 6 months.
e) Cost-benefit analysis of the complex intervention.
f) Quality of life at 1, 3 and 6 months using the validated Postpartum Quality of Life questionnaire [30] (APPENDIX-K).
g) Success of mothers in breastfeeding new babies exclusively, measured as the percent difference in 6-month breastfeeding exclusivity rates between the intervention and control groups in subsequent babies up to 5 years later.

Sample size calculation

We will consider the rate of exclusive breastfeeding at the end of six months in the control group to be 2%, which is the most recently reported rate from Lebanon [23]. We hypothesize that our complex intervention will increase the 6-month exclusivity rate to 12% in the intervention group (i.e. difference of 10%). Assuming a power of 90% and a type I error of 5%, the number of subjects needed per group will be 155 mothers, and the total sample size will be 310 mothers. To allow for a loss-to-follow up rate of 30%, the needed sample size will become = 310/(1-0.3) = 310/0.7 = 443 subjects. The number of SMs needed is 74 (1 SM for each 3 BFMi).

Data collection

Participants

Baseline: Age, socio-economic status, education, employment status, mother’s religion, baseline breastfeeding knowledge and attitude, parity, previous breastfeeding, baseline social support, study site, residence.

Month 1: Baby’s gender, mode of delivery, pediatrician’s gender, first feed at hospital, rooming in, breastfeeding status, baby’s illness visits (number, diagnosis, money spent on medicine and doctor’s fees), baby’s hospitalizations (number, diagnosis, cost), days lost from work due to baby’s illness, days lost from work due to mother’s illness, cost of formula feeds/week, cost of water used to prepare formula/week, mother’s non-routine doctor visits due to breastfeeding, cost of infant food/week, QOL.

Month 3: Breastfeeding status, baby’s illness visits (number, diagnosis, money spent on medicine and doctor’s fees), baby’s hospitalizations (number, diagnosis, cost), days lost from work due to baby’s illness, days lost from work due to mother’s illness, cost of formula feeds/week, cost of water used to prepare formula/week, mother’s non-routine doctor visits due to breastfeeding, cost of infant food/week, QOL.

Month 6: Breastfeeding status, baby’s illness visits (number, diagnosis, money spent on medicine and doctor’s fees), baby’s hospitalizations (number, diagnosis, cost), days lost from work due to baby’s illness, days lost from work due to mother’s illness, cost of formula feeds/week, cost of water used to prepare formula/week, mother’s non-routine doctor visits due to breastfeeding, cost of infant food/week, QOL, satisfaction of mother with mother-to-mother support system, breastfeeding knowledge and attitude of mother, in-depth interviews with BFMis.

Year 1: Breastfeeding status, baby's illness visits (number, diagnosis, money spent on medicine and doctor's fees), baby's hospitalizations (number, diagnosis, cost), days lost from work due to baby's illness, days lost from work due to mother's illness, cost of formula feeds/week, cost of water used to prepare formula/week, mother's non-routine doctor visits due to breastfeeding, cost of infant food/week.

Year 2: Breastfeeding status, baby's illness visits (number, diagnosis, money spent on medicine and doctor's fees), baby's hospitalizations (number, diagnosis, cost), days lost from work due to baby's illness, days lost from work due to mother's illness, cost of formula feeds/week, cost of water used to prepare formula/week, mother's non-routine doctor visits due to breastfeeding, cost of infant food/week, breastfeeding status of new baby.

Support Mothers

Baseline: Age, socio-economic status, education, employment status, religion, baseline breastfeeding knowledge and attitude, parity, previous breastfeeding.

At end of support activity of each participant: Activity log sheets, satisfaction with mother-to-mother support system.

Year 1: In-depth interviews with SMs.

Nurses

Baseline: age, socio-economic status, education, employment status, parity, previous breastfeeding.

Month 6: Satisfaction with the experience.

Statistical analysis

We will compare continuous variables using Student's *t* test and categorical variables using Chi square test. The relationship between breastfeeding exclusivity (outcome) and other variables (predictors) such as maternal age, study group (intervention/control), socio-economic status, maternal education, study site, pediatrician's gender, type of delivery, baby's gender, parity, previous breastfeeding, maternal employment, mother's religion, baseline breastfeeding knowledge and attitude, baseline social support will be explored in bivariate and multivariate analysis. Regression models will be built to adjust for possible confounding in the relationship between the dependent and the independent variables stated above. All analysis will be done on intention to treat basis. The Statistical Package for Social Sciences (SPSS) will be used for data management and analyses. A p-value of <0.05 will indicate statistical significance.

Ethical Considerations

The current proposal will be submitted to the Internal Review Boards of the American University of Beirut and Al-Sahel General Hospital for ethical approval. We have obtained the preliminary approval of the Administration and the collaborating physicians at Al-Sahel Hospital. All participant pregnant women and support mothers will be requested to give their written informed

consent prior to any study procedure. To secure confidentiality, all identifying information of participants, including name, medical and contact information as well as all data collected will be kept in a password protected computer that is kept secure, and in a locked cabinet in the office of Dr. Mona Nabulsi. Data access will be limited to the principal investigators and project coordinator working directly on the study. All data will be destroyed responsibly after five years from publication of the main trial and all sub-studies emanating from it.

To ensure appropriate medical and /or psychiatric care is provided to those mothers who need it, an ongoing evaluation process will take place with referral to appropriate services after physician notification and evaluation.

SM training will emphasize empathy and encouragement in order to ensure BFMis do not feel pressured to continue breastfeeding if they do not wish to do so. BFMis who fail to continue exclusive breastfeeding will continue to receive positive messages and get support if they so wish. This will continue until six months postpartum or until the BFMi decides to stop, whichever comes first.

Budget

**ITEM**		**Year 1**	**Year 2**	**Justification**
**Personnel**	Project coordinator to manage study procedures	$ 13,680	$ 13,680	MS level, Full-time basis @ $1,140/month for 2 years.
	Research assistant to recruit from AUBMC site	$ 10,944	$ 10,944	BS level, Full-time basis @ $912/month for 2 years.
	Research assistant to recruit from Sahel Hospital site	$ 10,944	$ 10,944	BS level, Full-time basis @ $912/month for 2 years.
	Research assistant for the qualitative interviews	$ 10,260	_____	MS level, Full-time basis @ $1,140/month for 9 months.
	Pediatrics nurse for participants from AUBMC	$ 10,944	$ 5,472	BS level, Full-time basis @ $912/month for 18 months.
	Pediatrics nurse for Sahel Hospital participants	$ 10,944	$ 5,472	BS level, Full-time basis @ $912/month for 18 months.
**Training workshops**<br><br>**(Training material, exams, and**	Two training workshops for project coordinator and research assistants	$ 200	____	Two workshops to train on work plan and data collection. Cost includes training material, snack & refreshments.
	Workshop for the nurses	$ 1,060	____	One WHO training workshop on breastfeeding plus fee for the International Lactation Consultant Certification Exam

**refreshments)**				@ $ 530/nurse, for 2 nurses.
	Workshops for the support mothers	$ 4,440	——	Two training workshops for each support mother @ $ 60/mother; total of 74 support mothers.
**Local transport**	Research assistant interview visits	$ 500	——	50 visits for the qualitative interviews @ $ 10/visit.
	Nurses interview visits	$ 11,100	$ 11,100	10 home visits/breastfeeding mother@ $ 10/visit; for 222 mothers.
	Support mothers interview visits	$ 6,660	$ 6,660	6 home visits/breastfeeding mother@ $ 10/visit; for 222 mothers.
**Dissemination**	Video	$ 10,000	——	Video production.
	Breastfeeding education pamphlet	$ 4,500	——	Printing of pamphlets @ $15/pamphlet.
**Supplies and other direct costs**	Phone cards for nurses and support mothers	$ 6,000	$ 3,040	226 cards (2 cards/research assistant; 3 cards/support mother) @ $ 40/card.
	Flyers, questionnaires and data collection sheets	$ 1,000	——	Paper & printing cost.
**Subtotal**		$ 113,176	$ 67,312	
**Overhead @ 20%**		$ 22,635	$ 13,463	
**Grand total**		$ 135,811	$ 80,774	
**Total for 2 years**	**$ 216,585**			

Studies emanating from this project

1. Sub-study 1: The mother-to-mother breastfeeding support pilot study (led by Dr. Haya Hamadeh); Study approved by the IRB.
2. Sub-study 2: Development and validation of the Arabic breastfeeding knowledge, attitude and behavior (B-KAB) questionnaire (led by Drs. Lama Charafeddine and Durriyah Sinno)
3. Sub-study 3: Assessment of the impact of the complex breastfeeding intervention on knowledge and attitudes of breastfeeding mothers and their families (led by Drs. Durriyah Sinno and Lama Charafeddine, and Mrs. Saadieh Masri)

4. Sub-study 4: A qualitative study on the experiences of breastfeeding mothers and support mothers participating in the complex breastfeeding promotion and support intervention trial (led by Dr. Tamar Kabakian).
5. Sub-study 5: Professional nurses experiences and satisfaction with the breastfeeding promotion and support intervention (led by Mrs. Saadieh Masri and Dr. Durriyah Sinno)
6. Sub-study 6: Impact of the complex breastfeeding intervention on the quality of life of participating mothers (led by Drs. Haya Hamadeh, Tamar Kabakian and Mona Nabulsi)
7. Sub-study 7: Cost-benefit analysis of the complex breastfeeding intervention (led by Dr. Nadine Yehya)
8. Sub-study 8: Development and validation of the Arabic Postpartum Quality of Life Questionnaire (led by Drs Hani Tamim and Mona Nabulsi)

Time Commitments and Funds Available

Dr. Mona Nabulsi will dedicate 30% of her time to supervise this project.

Dr. Haya Hamadeh will dedicate 25% of her time to supervise this project.

The pilot feasibility study (Sub-study 1) received recently funding from the seed fund available to Dr Haya Hamadeh. Otherwise, there are no funds available yet for this project.

Co-investigators

Dr. Lama Charafeddine (10%)

Dr. Durriyah Sinno (10%)

Dr. Tamar Kabakian (5%)

Dr. Nadine Yehya (5%)

Dr. Hani Tamim (5%)

Mrs. Saadieh Sidani (10%)

Timeline

	Oct.-Dec. 2013	Jan.-Dec. 2014	Jan.-June 2015	Jul.-Oct. 2015	Nov. 2015-Oct. 2018
**Hiring & training of staff**	X				
**Printing of pamphlets & data collection sheets**	X				
**Production of Video**	x				
**Development & validation of Arabic questionnaires**	X				
**Subject recruitment**		X	X		
**Training of SMs**		X			
**Data collection**		X	X	X	X
**Data entry/analysis**		X	X	X	X

References

1. UNICEF, Ministry of Public Health, Lebanese Republic: *National Perinatal Survey*. 1999- 2000, 10.

2. Batal M, Boulghourian C: **Breastfeeding initiation and duration in Lebanon: are the hospitals "Mother Friendly"?** *J Pediatr Nurs* 2005, **20**: 53-59.

3. Ministry of Social Affairs: *Pap Child Survey*. 1996.

4. Batal M, Boulghourian C, Abdallah A, Afifi R: **Breast-feeding and feeding practices in a developing country: a national survey in Lebanon.** *Public Health Nutr* 2006, **9**: 313-319.

5. **World Health Organization (WHO): Global data bank on infant and young child feeding**. [http://www.who.int/nutrition/databases/infantfeeding/countries/lbn.pdf]

6. Chakar Rabay H, Sokhn M, Azar M: **Breast-feeding practice in a localized area of Beirut**. *LMJ* 1997, **45**: 84-89.

7. Ip S, Chung M, Raman G, Chew P, Magula N, DeVine D, Trikalinos T, Lau J: **Breastfeeding and maternal and infant health outcomes in developed countries.** *Evid Rep Technol Assess* 2007, **153**:1-186.

8. Kramer MS, Chalmers B, Hodnett ED, Sevkovskaya Z, Dzikovich I, Shapiro S, Collet JP, Vanilovich I, Mezen I, Ducruet T, Shishko G, Zubovich V, Mknuik D, Gluchanina E, Dombrovskiy V, Ustinovitch A, Kot T, Bogdanovitch N, Ovchinikova L, Helsing E: PROBIT Study Group (Promotion of Breastfeeding Intervention Trial): **Promotion of breastfeeding intervention trial (PROBIT): a randomized trial in the Republic of Belarus.** *JAMA* 2001, **285**:413–20.

9. Kramer MS: **"Breast is best": the evidence.** *Early Hum Dev* 2010, **86**:729-732.

10. Molbak K, Gottschau A, Aaby P, Hojlyng N, Ingholt L, Da Silva APJ: **Prolonged breastfeeding, diarrhoeal disease, and survival of children in Guinea-Bissau.** *BMJ* 1994, **308**:1403–6.

11. WHO Collaborative Study Team on the Role of Breastfeeding on the Prevention of Infant Mortality: **Effect of breastfeeding on infant and child mortality due to infectious diseases in less developed countries: a pooled analysis.** *Lancet* 2001, **355**:451-455.

12. Gareth Jones, Richard W Steketee, Robert E Black, Zulfiqar A Bhutta, Saul S Morris, and the Bellagio Child Survival Study Group: **How many child deaths can we prevent this year?** *Lancet* 2003, 362: 65-71.

13. Al-Sahab B, Tamim H, Mumtaz G, Khawaja M, Khojali M, Afifi R, Nassif Y, Yunis KA: For the National Collaborative Perinatal Neonatal Network (NCPNN). **Predictors of breastfeeding in a developing country: results of a prospective cohort study**. *Public Health Nutr* 2008, **11**:1350-1356.

14. Nabulsi M. **Why are breastfeeding rates low in Lebanon? A qualitative study**. *BMC Pediatrics* 2011 Aug 30;**11**:75.

15. Britton C, McCormick FM, Renfrew MJ, Wade A, King SE. **Support for breastfeeding mothers.** *Cochrane Database of Systematic Reviews* 2007, Issue 1. Art. No.: CD001141.pub 3.

16. Chung M, Ip S, Yu W, Raman G, Trikalinos T, DeVine D, Lau J. **Interventions in Primary Care to Promote Breastfeeding: A Systematic Review**. *Evidence Syntheses* 2008,66 Report No.: 09-05126-EF-1.

17. Wade D, Haining S, Day A. **Breastfeeding peer support: are there additional benefits?** *Community Pract* 2009, 82 (12): 30-33.

18. Kate Jolly et al. **Systematic review of peer support for breastfeeding continuation: meta-regression analysis of the effect of setting, intensity, and timing**. *BMJ* 2012, 344: d82-87.

19. Jennifer Hall. **Effective community-based interventions to improve exclusive breastfeeding at four to six months in low and middle-income countries: a systematic review of randomised controlled trials**. *Midwifery* 2011, 27: 497-502

20. Kaern D'Entremont et al. Mother to Mother: **Creating a Breastfeeding Support Line in Your Community**. Crown Copyright, Province of Nova Scotia 2007.

21. Renfrew MJ, McCormick FM, Wade A, et al. **Support for healthy breastfeeding mothers with healthy term babies**. *Cochrane Database of Systematic Reviews* 2012 May 16;5:CD001141.

22. Dyson L, McCormick FM, Renfrew MJ. **Interventions for promoting the initiation of breastfeeding.** *Cochrane Database of Systematic Reviews* 2005, Issue 2. Art. No.: CDD001688. DOI: 10.1002/14651858.CD001688.pub2.

23. UNICEF, Central Administration of Statistics, Beirut, Lebanon. *Multiple indicator cluster survey final report*. 2009.

24. Heany CA, Israel BA. *Social networks and social support*. In: Glanz K, Lewis FM, Rimer BK (edt) Health behavior and health education: theory, research and practice. Jossey-Bass Publishers: San Francisco, 1997.

25. De La Mora A, Russel DW. **The Iowa infant feeding attitude scale: analysis of reliability and validity**. J Appl Soc Psychol 1999, 29:2362-2380.

26. Nommsen-Rivers LA, Dewey KG. **Development and validation of the infant feeding intentions scale**. *Matern Child Health J* 2009, 13:334–342.

27. Libbus MK. **Perspectives of common breastfeeding situations: a known group comparison**. *J Hum Lact* 1992, 8:199-203.

28. Libbus MK. **Breastfeeding attitudes in a sample of Spanish-speaking Hispanic American women**. *J Hum Lact* 2000, 16:216-220.

29. Grossman LK, Harter C, Sachs L, Kay A. **The effect of postpartum lactation counseling on the duration of breast-feeding in low-income women**. *Am J Dis Child* 1990, 144:471-474.

30. Hill PD, Aldag JC, Hekel B, Riner G, Bloomfield P. **Maternal postpartum quality of life questionnaire.** *J Nurs Measurement* 2006, 14:205-220.
